# Analyzing Fall Incidents and Fall-Related Injuries in Wisconsin Assisted Living Communities

**DOI:** 10.1093/geroni/igaf122.3458

**Published:** 2025-12-31

**Authors:** Sohee Kim, James Ford

**Affiliations:** University of Wisconsin - Madison, Madison, Wisconsin, United States; University of Wisconsin–Madison, Madison, Wisconsin, United States

## Abstract

This study examines trends in fall incidence and fall-related injuries in Wisconsin assisted living communities (ALCs) from 2017 to 2022. It aims to identify differences in fall trends among three ALC types—Adult Family Homes (AFHs), Community-Based Residential Facilities (CBRFs), and Residential Care Apartment Complexes (RCACs)—and to propose targeted interventions for reducing falls and enhancing resident safety. The study analyzes data from the Wisconsin Coalition for Collaborative Excellence in Assisted Living (WCCEAL), incorporating fall reports from ALCs across the state. The dataset includes quarterly records from 2017 to 2022, covering facilities that voluntarily tracked and shared fall and hospitalization data. Falls and fall-related injury data were evaluated using incidence rates per 1,000 resident days, stratified by facility type. Facility characteristics, including occupancy, primary resident population, and location, were analyzed to identify patterns in falls. The study used chi-square tests to compare sample and non-sample cohorts. Overall, the total number of falls in ALCs increased during the study period, whereas fall-related injuries decreased. CBRFs and RCACs showed substantial increases in fall incidence rates, while AFHs showed a declining trend. Findings emphasize the need for customized fall prevention interventions across different ALC types. AFHs may benefit from individualized interventions, CBRFs from staff training and structured fall protocols, and RCACs from self-management strategies like emergency response systems. Standardizing fall reporting practices and enhancing fall prevention policies at both the state and facility levels could improve resident safety and reduce fall-related injuries.

